# Substrate specificity and regioselectivity of fungal AA9 lytic polysaccharide monooxygenases secreted by *Podospora anserina*

**DOI:** 10.1186/s13068-015-0274-3

**Published:** 2015-06-20

**Authors:** Chloé Bennati-Granier, Sona Garajova, Charlotte Champion, Sacha Grisel, Mireille Haon, Simeng Zhou, Mathieu Fanuel, David Ropartz, Hélène Rogniaux, Isabelle Gimbert, Eric Record, Jean-Guy Berrin

**Affiliations:** INRA, UMR1163 Biodiversité et Biotechnologie Fongiques, Faculté des Sciences de Luminy, ESIL Polytech, F-13288 Marseille, France; Polytech Marseille, Aix Marseille Université, F-13288 Marseille, France; Institute of Chemistry, Slovak Academy of Sciences, 84538 Bratislava, Slovakia; INRA, Plateforme BIBS, Unité de Recherche Biopolymères, Interactions, Assemblages, 44316 Nantes, France

**Keywords:** AA9, LPMO, Cellobiose dehydrogenase, Oxidized cello-oligosaccharides, Cellulose, Hemicellulose, Oxidative cleavage, Lignocellulose, Biomass, Biorefinery

## Abstract

**Background:**

The understanding of enzymatic polysaccharide degradation has progressed intensely in the past few years with the identification of a new class of fungal-secreted enzymes, the lytic polysaccharide monooxygenases (LPMOs) that enhance cellulose conversion. In the fungal kingdom, saprotrophic fungi display a high number of genes encoding LPMOs from family AA9 but the functional relevance of this redundancy is not fully understood.

**Results:**

In this study, we investigated a set of AA9 LPMOs identified in the secretomes of the coprophilous ascomycete *Podospora anserina*, a biomass degrader of recalcitrant substrates. Their activity was assayed on cellulose in synergy with the cellobiose dehydrogenase from the same organism. We showed that the total release of oxidized oligosaccharides from cellulose was higher for *Pa*LPMO9A, *Pa*LPMO9E, and *Pa*LPMO9H that harbored a carbohydrate-binding module from the family CBM1. Investigation of their regioselective mode of action revealed that *Pa*LPMO9A and *Pa*LPMO9H oxidatively cleaved at both C1 and C4 positions while *Pa*LPMO9E released only C1-oxidized products. Rapid cleavage of cellulose was observed using *Pa*LPMO9H that was the most versatile in terms of substrate specificity as it also displayed activity on cello-oligosaccharides and β-(1,4)-linked hemicellulose polysaccharides (e.g., xyloglucan, glucomannan).

**Conclusions:**

This study provides insights into the mode of cleavage and substrate specificities of fungal AA9 LPMOs that will facilitate their application for the development of future biorefineries.

**Electronic supplementary material:**

The online version of this article (doi:10.1186/s13068-015-0274-3) contains supplementary material, which is available to authorized users.

## Background

Lignocellulosic biomass is a high-potential renewable resource for the production of 2nd-generation biofuels and platform molecules for the chemical industry. The natural resistance of plant cell wall to microbial and enzymatic deconstruction, collectively known as “biomass recalcitrance”, is largely responsible for the high cost of industrial processes [1]. Although plant biomass degradation by fungi has been studied extensively, our knowledge of the enzyme systems used to degrade cellulose has changed dramatically in the last 5 years. Indeed, a new class of secreted enzymes known as lytic polysaccharide monooxygenases (LPMOs) was identified due to its “boosting effect” on enzymatic polysaccharide conversion [2, 3]. Intensive efforts have started to unveil their function in the oxidative degradation of cellulose [4–8] and other plant polysaccharides, i.e., hemicellulose [9] and starch [10, 11].

All LPMOs share a common structural fold with a flat surface where binding with the substrate occurs mostly via stacking interactions with planar aromatic residues. A type II copper ion exposed at the surface is coordinated by the nitrogen atoms of two highly conserved histidine residues, one of which corresponds to the N-terminal histidine. Analysis of LPMOs’ reaction products showed that they produced C1- and/or C4-oxidized oligomers. According to the preferred site of oxidation, three classes of AA9 LPMOs were described, type 1 and type 2 oxidizing at the C1 and the C4, respectively, and type 3 oxidizing at both the C1 and C4 carbon atoms of glucose [8, 12, 13]. C6 oxidation has been suggested for *Thermoascus aurantiacus Ta*GH61A [7] and *Podospora anserina Pa*GH61B [14] although it is a matter of debate since the 6-hexodialdoses have the same molecular weight as that of the corresponding 4-ketoaldioses.

LPMOs are classified into four auxiliary activity (AA) families, AA9 (formerly GH61), AA10 (formerly CBM33), AA11, and AA13 of the Carbohydrate-Active enZyme database [15, 16] (CAZy; http://www.cazy.org). The AA10 family contains mainly enzymes of bacterial and viral origin that cleave cellulose and chitin mostly at the C1 position [17, 18]. The LPMOs classified in the AA11 and AA13 families, respectively, cleave chitin and starch and share important structural features with the two previously characterized families [10, 11, 18].

The oxidative cleavage performed by LPMOs is occurring in the presence of small redox-active molecules such as ascorbic acid, reduced glutathione, or gallate [2, 4, 7, 17]. The peculiarity of fungal AA9 LPMOs is their action in concert with cellobiose dehydrogenases (CDHs) since their association resulted in redox-mediated glycosidic bond cleavage in cellulose, assuming a key role of this oxidative system in fungi [5, 6, 8, 14, 19]. All known CDHs fall into two related subgroups. Class I members are represented by sequences from basidiomycetes whereas class II comprises longer, more complex sequences from ascomycete fungi [20]. The effectiveness of LPMO/CDH synergy depends on enzyme concentrations and the type of substrate used [14].

The family AA9 comprises about 300 fungal members widely distributed in the genomes of most ascomycetes and basidiomycetes. A striking feature is the extreme expansion in genes encoding AA9s observed in some genomes, which can reach more than 30 homologous gene models per species. A total of 33 candidate AA9s were assigned in *P. anserina* [16, 21]. Only two (*Pa*GH61A and *Pa*GH61B) were functionally characterized for their potential to degrade cellulose oxidatively [14]. Therefore, this redundancy raises the question of the functional relevance at the organism level, i.e., functional redundancy or functional diversification or fine-tuned regulation of alternative genes and/or adaptations to the degradation of the substrates. AA9 LPMOs are frequently multimodular bearing a CBM1 at their C terminus. Post-genomic analyses of saprophytic fungi have shown that AA9 LPMOs’ isoforms are specifically secreted during degradation of lignocellulosic substrates [22–27]. In this study, we focused on AA9 LPMOs identified in the secretome of *P. anserina* that was shown to significantly improve the saccharification yield of steam-exploded wheat straw [26]. The specificity and regioselectivity of these fungal AA9 LPMOs for the degradation of plant cell wall carbohydrates were investigated using complementary approaches.

## Results

### Heterologous expression of five AA9 LPMOs and one CDH from *P. anserina*

**Fig. 1:**
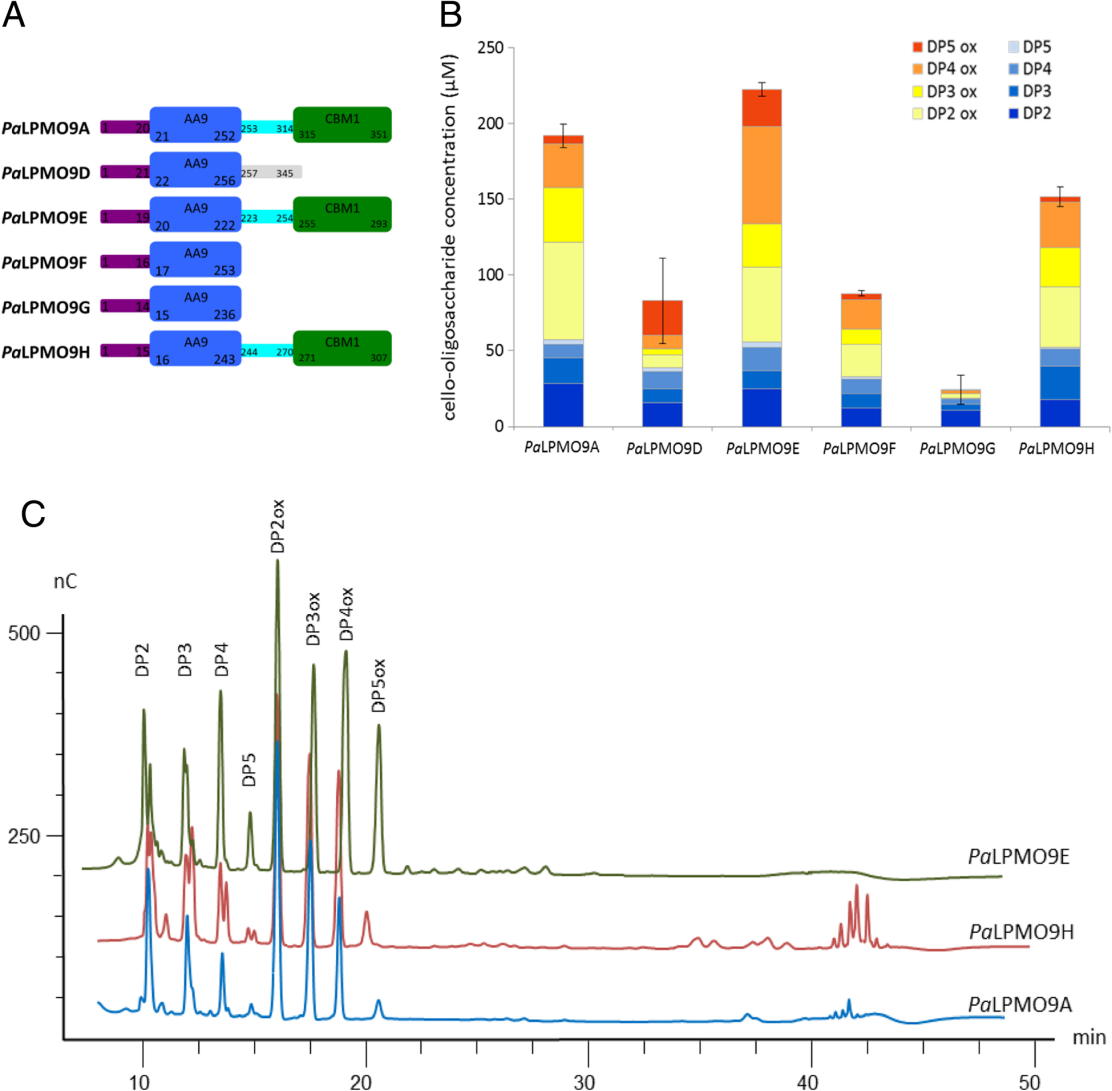
Oxidative cleavage of cellulose by *P. anserina* AA9 LPMOs. **a** Schematic representation of the modularity of *P. anserina* AA9 LPMOs based on CAZy annotation. **b** Quantification by HPAEC analysis of the soluble sugars released by 4.4 μM *Pa*LPMO and 1.2 U.ml^−1^ of *Pa*CDHB, at 50 °C for 16 h. The concentration of each sugar was determined by integration of the peak area and comparison with a standard curve. Values are the mean of three biological replicates (*n* = 3). *Error bars* correspond to one cumulated SD (error bar = ±SDtot; with SDtot = √(SD_1_^2^ + SD_2_^2^ + …). **c** HPAEC chromatograms showing products generated from cellulose with *Pa*LPMO9A, *Pa*LPMO9H, and *Pa*LPMO9E. The peak annotations are based on comparison with oligosaccharides standards oxidized at the C1 position (DP2ox-DP5 ox)

In order to give insights into the functional relevance of *P. anserina* LPMOs, we selected seven AA9 LPMOs and one CDH, i.e., *Pa*LPMO9A [14], *Pa*LPMOC (protein ID CAP68173), *Pa*LPMO9D (protein ID CAP66744), *Pa*LPMO9E (protein ID CAP67740), *Pa*LPMO9F (protein ID CAP71839), *Pa*LPMO9G (protein ID CAP73072), *Pa*LPMO9H (protein ID CAP61476), and *Pa*CDHB (protein ID CAP61651) based on our previous analysis of *P. anserina* secretomes [26]. The *Pa*CDHB was selected because it was more abundant in the secretomes compared to *Pa*CDHA, which was previously characterized [28]. In all *P. anserina* LPMOs selected, the three amino acids (two histidines with one at the first position and one tyrosine) involved in copper coordination were strictly conserved (Additional file 1: Figure S1). However, the *P. anserina* LPMOs selected are quite diverse in sequence with identities ranging from 23 to 53 % within the main catalytic domain (Additional file 1: Table S1). *Pa*LPMO9A, *Pa*LPMO9E, and *Pa*LPMO9H are multimodular enzymes with a carbohydrate-binding module from the family CBM1 at their C terminus (Fig. 1a).

Because the strictly conserved N-terminal histidine residue is essential for their function, the *P. anserina lpmo* genes, codon optimized for expression in *Pichia pastoris*, were inserted into the pPICZαA expression plasmid immediately after the sequence encoding the signal peptide. Following induction of selected *P. pastoris* transformants, all the *P. anserina* AA9s (except *Pa*LPMO9C) were successfully produced and purified to homogeneity. Electrophoretic analysis revealed that purified *Pa*LPMO9s displayed apparent molecular masses that were higher than the theoretical ones (Additional file 1: Figure S2). This might be partly due to O- and N-glycosylation that are predicted to be abundant in all of the *Pa*LPMO9s, especially the ones bearing serine/threonine-rich linker regions between the catalytic and the CBM1 modules that contain multiple glycosylation sites as already observed in other modular fungal CAZymes [29].

A peculiarity of fungal AA9 LPMOs is the methylation of their N-terminal histidine at the imidazole Nε2 [18, 27]. To investigate whether *Pa*LPMO9s harbored this unusual post-translational modification, mass spectrometry (MS) analyses on tryptic digests of *Pa*LPMO9E and *Pa*LPMO9H were performed. MS spectra supported that the strictly conserved N-terminal histidine residue was not methylated (Additional file 1: Table S2).

To assess the functionality of these copper monooxygenases, we evaluated their capacity to produce H_2_O_2_ when activated by ascorbate in the absence of a carbohydrate substrate. Although H_2_O_2_ production is not the intended natural enzyme reaction, it can be used to calculate specific activities. Specific activities measured with enzymes purified to homogeneity were ranging from 0.01 U.g^−1^ (*Pa*LPMO9A) to 1.95 U.g^−1^ (*Pa*LPMO9E) (Table 1). No significant H_2_O_2_ production was detected for *Pa*LPMO9G.

**Table 1:** Biochemical and enzymatic characteristics of the enzymes used in this study

Enzymes	GenBank ID	Modularity	Predicted function	SA^a^ (U.g^−1^)	Competitive inhibition assay (% of residual activity)	
					PASC	CMC
*Pa*CDHB	CAP61651	AA3_1-AA8	CDH	8.6	N.D.	N.D.
*Pa*LPMO9A	CAP73254	AA9-CBM1	LPMO	0.010 ± 0.001	7.6 ± 1.1	97.9 ± 2.1
*Pa*LPMO9D	CAP66744	AA9	LPMO	0.083 ± 0.005	75.1 ± 7.5	73.2 ± 8.5
*Pa*LPMO9E	CAP67740	AA9-CBM1	LPMO	1.95 ± 0.04	18.4 ± 4.2	47.0 ± 1.3
*Pa*LPMO9F	CAP71839	AA9	LPMO	0.028 ± 0.003	16.9 ± 1.9	100.0 ± 4.8
*Pa*LPMO9G	CAP73072	AA9	LPMO	n.d.	N.D.	N.D.
*Pa*LPMO9H	CAP61476	AA9-CBM1	LPMO	0.42 ± 0.03	7.2 ± 1.4	6.1 ± 0.8

*n.d*. no production of H_2_O_2_ detected using the Amplex Red assay, *N.D*. not determined

^a^Specific activity was measured using DCPIP (pH 5, 30 °C) for *Pa*CDHB and the Amplex Red assay for *Pa*LPMOs (pH 6, 30 °C)

In parallel, *Pa*CDHB was also heterologously expressed in *P. pastoris*. It exhibited a specific activity of 8.6 U.mg^−1^ on cellobiose when dichlorophenol indophenol (DCPIP) was used as electron acceptor and was able to successfully oxidize cello-oligosaccharides from DP2 to DP6 yielding the corresponding aldonic acid oligosaccharides (oxidized at C1) that were used as standards for the present study.

### LPMOs bearing a CBM1 module display higher cellulose degradation capabilities

To check their functionality, *Pa*LPMO9s were tested for their ability to produce H_2_O_2_ in the presence of a range of cellulosic derivatives using the Amplex Red assay (see Material and Methods) described by Isaksen et al. [30] and Kittl et al. [31]. Indeed, the H_2_O_2_ production of LPMOs in the absence of carbohydrate substrate is a futile reaction that is less likely to happen when the substrate is available. For all *Pa*LPMO9s tested, the production of H_2_O_2_ decreased in the presence of phosphoric acid swollen cellulose (PASC) and carboxymethyl cellulose (CMC). Except *Pa*LPMO9D, the presence of PASC almost completely reduced the H_2_O_2_ production while CMC significantly reduced H_2_O_2_ production for *Pa*LPMO9E and *Pa*LPMO9H (Table 1).

*Pa*LPMOs were further assayed for their ability to cleave cellulose in the presence of *Pa*CDHB. High performance anion exchange chromatography (HPAEC) analysis, following the method developed for the detection of both non-oxidized and oxidized species [32], indicated that the combination of *Pa*LPMO9s with *Pa*CDHB released a mixture of non-oxidized and oxidized soluble cello-oligosaccharides that were quantified based on standards. The degree of polymerization (DP) ranged from DP2 to DP5 for non-oxidized oligosaccharides and from DP2ox to DP5ox for the oxidized products (Fig. 1b, c). The total release of non-oxidized and oxidized oligosaccharides was more efficient for *Pa*LPMO9A, *Pa*LPMO9E, and *Pa*LPMO9H that possess a CBM1 module. Analyses of chromatograms indicated differences in terms of oxidized products (Fig. 1c). For instance, *Pa*LPMO9E released oligosaccharides corresponding to aldonic acid oligosaccharides (C1-oxidized) while for *Pa*LPMO9A and *Pa*LPMO9H, peaks eluting later were also observed (Fig. 1c).

#### Oxidative regioselectivity of *Pa*LPMO9A, *Pa*LPMO9E, and *Pa*LPMO9H

**Fig. 2:**
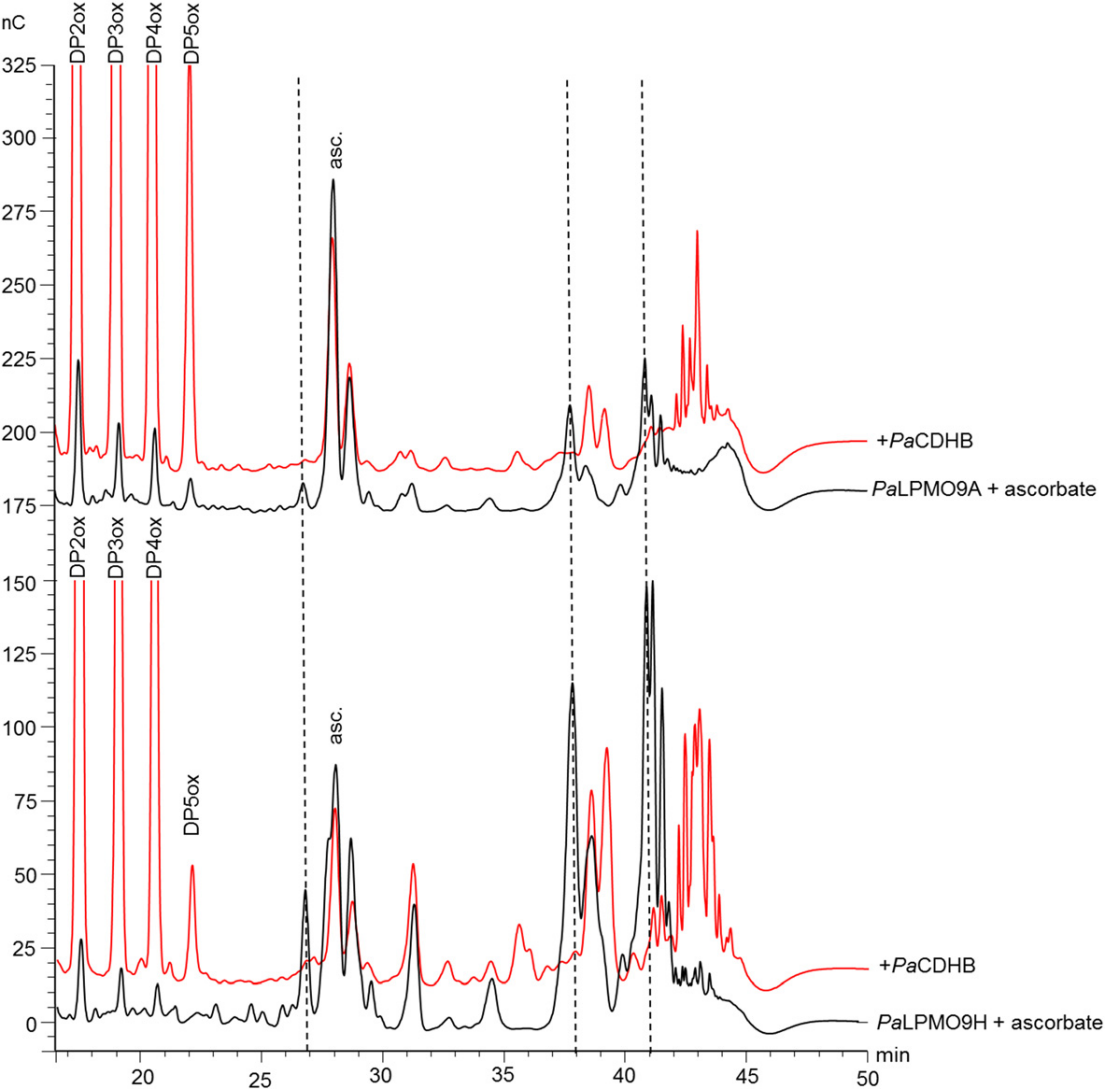
Analysis of degradation products generated by *Pa*LPMO9A and *Pa*LPMO9H. The HPAEC chromatograms of the oligosaccharides released upon degradation of 0.1 % PASC with 4.4 μM *Pa*LPMO in the presence of 1 mM ascorbate, at 50 °C for 16 h (in *black*) followed by the incubation with 1.2 U.ml^−1^ of *Pa*CDHB at 50 °C for 8 h (in *red*). The peak annotations are based on comparison with oligosaccharides standards oxidized at the C1 position (DP2ox-DP5ox). Coelution of DP1ox with DP3 and DP2ox with DP6 was observed. Peaks eluting at 27, 38, and 41 min are annotated with *dotted lines*

To determine the nature of oxidation of *Pa*LPMO9A, *Pa*LPMO9E, and *Pa*LPMO9H, we performed sequential treatments of cellulose by LPMOs in the presence of ascorbate followed by addition of *Pa*CDHB, which oxidizes only at the reducing end (C1 carbon of the glucose unit) of cellobiose and longer cello-oligosaccharides. In the case of *Pa*LPMO9E, only C1-oxidized oligosaccharides were detected in both the ascorbate and *Pa*CDHB conditions (not shown). In the case of *Pa*LPMO9A and *Pa*LPMO9H, the ascorbate condition yielded production of C1-oxidized oligosaccharides (DP2–DP4) as well as other oxidized species observed at 27, 38, and 41 min (Fig. 2). For both *Pa*LPMO9A and *Pa*LPMO9H, addition of *Pa*CDHB led to an increase of C1-oxidized oligosaccharides and to a clear shift of products eluting after 25 min. Indeed, oxidized species at 27, 38, and 41 min disappeared with the concomitant apparition of new peaks at 39 min and around 42–44 min, which may correspond to a C1–C4-double-oxidized DP2 and longer double-oxidized products, respectively, based on the analysis of Isaksen et al. [30]

**Fig. 3:**
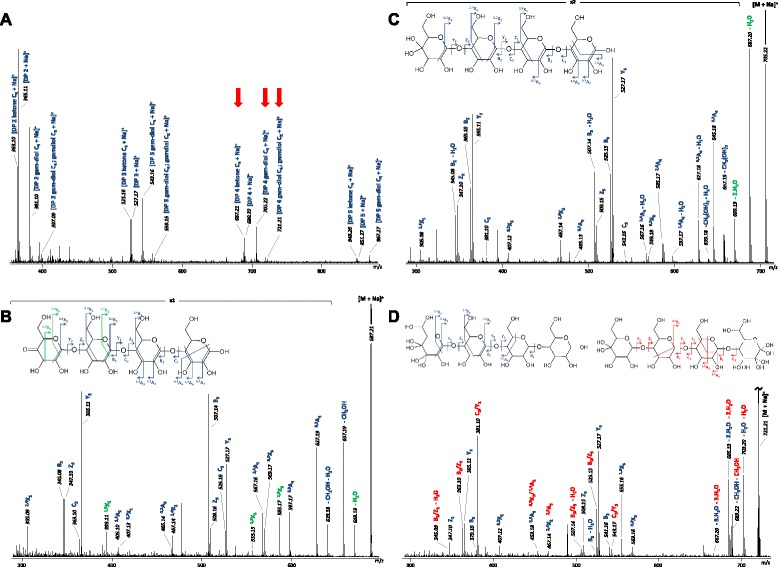
Mass spectrometry analysis of degradation products generated by *Pa*LPMO9H. **a** Analysis was performed after 16 h of cellulose degradation. The *main panel* shows the full spectrum of sample with peaks corresponding to native and oxidized cello-oligosaccharides. Fragmented peaks are indicated by *arrows*. The panels below **b**, **c**, and **d** show the DP4 peaks with *m*/*z* value of 687.21, 705.22, and 721.21, respectively, that were fragmented using ESI MS. The oxidized oligosaccharides product species are represented in panels **b**, **c**, and **d** based on the fragmentation patterns. In panel **d**, the different product species corresponding to the fragmentation pattern are indicated by *blue* and *red dotted bonds*

As no C4-oxidized standards are available, mass spectrometry was used to confirm the position of oxidation. Analysis of the product mixture generated from PASC with *Pa*LPMO9H confirmed the presence of DP2- to DP5-oxidized and non-oxidized cello-oligosaccharides with products potentially corresponding to a ketone or gem-diol at the non-reducing end (*m*/*z* −2 or *m*/*z* +16, respectively) and to a lesser extent double-oxidized products (*m*/*z* +32) (Fig. 3a). To confirm the position of oxidation generated by *Pa*LPMO9H, electrospray ionisation (ESI) MS/MS was performed on DP4 mono-oxidized product species (ions at *m*/*z* +687 and *m*/*z* +705, Fig. 3b, c). Interpretation of the fragmentation spectra was based on the previous observation by Isaksen et al. [30] that a gem-diol form at the non-reducing end (C4 position) leads to a double loss of water. This double loss is very clearly observed on the MS2 spectrum of the species at *m*/*z* 705.22 (Fig. 3c), suggesting that this species corresponds to a gem-diol form at the non-reducing end. In contrast, a single water loss is observed on Fig. 3b (*m*/*z* +687), consistent with a ketone form at the C4 position of the non-reducing end, in addition to several characteristic fragment ions supporting this ketone structure (^2,5^X_3_, ^1,5^X_3_, ^1,5^X_2_). Figure 3d displays the MS2 spectrum recorded for the species at *m*/*z* +721.21. Based on the mass accuracy of the instrument, this mass was unequivocally attributed to the sodiated ion of the doubly oxidized DP4. Again, the fragmentation pattern was interpreted following the statements of Isaksen et al. [30], as well as by the observation of some specific fragments. This has led us to propose two structures presumably present as a mixture: in the first one, the two oxidations are brought by the non-reducing end. This is evidenced by the two ions at *m*/*z* +527.17 (Y_3_) and *m*/*z* +555.16 (^1,5^X_3_), indicating three consecutive non-oxidized glucose units in this structure, thereby suggesting that the fourth one is doubly oxidized. The specific fragment at *m*/*z* +555.16 (^1,5^X_3_) further indicates that the doubly oxidized unit is the glucose at the non-reducing end. Some fragment ions of the spectrum cannot arise from the previous form and indicate the presence of a second structure. We propose that this structure corresponds to the DP4 in which one oxidation is brought by the non-reducing end while the second one is located at the reducing end. This is supported for example by the ions at *m*/*z* +525.15 (B_3_) and *m*/*z* +543.17 (C_3_), which masses correspond to two consecutive non-modified glucose units and one oxidized glucose unit. Note, however, that the exact positioning of the oxidation at the reducing end could not be ascertained between the C1, C2, C3, or C6 positions. A similar mass spectrometry analysis of the products released from PASC using the *Pa*LPMO9E was performed. It revealed only the presence of C1-oxidized species (Additional file 1: Figure S3).

In conclusion, product analyses indicate that *Pa*LPMO9A and *Pa*LPMO9H cleaved cellulose at both the C1 and C4 glycosidic positions while *Pa*LPMO9E was specific for the C1 position only.

#### Time-course monitoring of cellulose cleavage

**Fig. 4:**
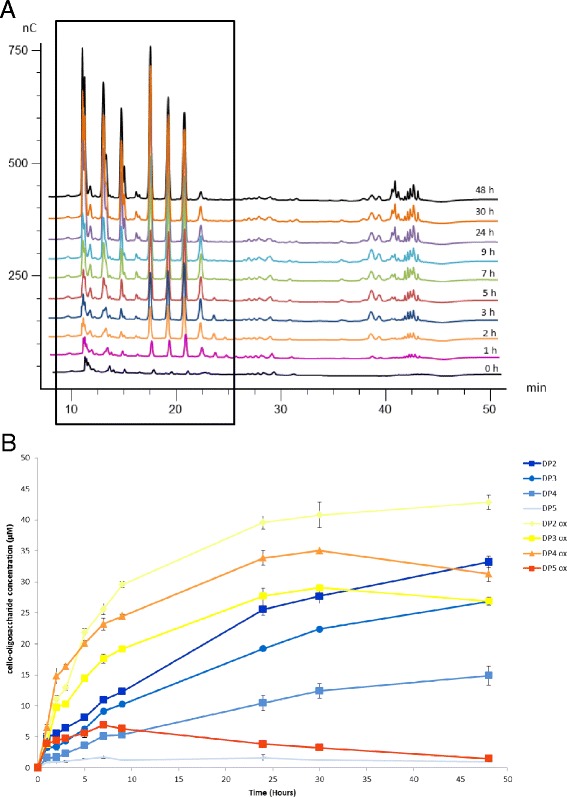
Time-course analysis of the products released from cellulose by *Pa*LPMO9H. **a** The HPAEC chromatograms show products generated from cellulose with 4.4 μM *Pa*LPMO9H and 1.2 U.ml^−1^ of *Pa*CDHB, at 50 °C for 1, 2, 3, 5, 7, 9, 24, 30, and 48 h of incubation. **b** A quantification by HPAEC analysis of the soluble sugars (aldonic acid and non-oxidized oligosaccharides) released by *Pa*LPMO9H over time has been conducted. The concentration of each sugar was determined by integration of the peak area and comparison with a standard curve. Values are the mean of three biological replicates (*n* = 3). *Error bars* correspond to one cumulated SD (error bar = ±SDtot; with SDtot = √(SD_1_^2^ + SD_2_^2^ + …)

Fungal members of the GH61 family have been described for many years as “weak endoglucanases” [33, 34] as the activity was several orders of magnitude lower than what had been observed in other endoglucanases. Since the discovery of the functional nature of AA9 LPMOs, their enzymatic activity is still described as slow for the release of soluble products [4, 12, 35] and therefore, their activity is usually measured using long-lasting incubations (overnight incubations). Using *Pa*LPMO9H, we followed over time the release of soluble oligosaccharides (oxidized and non-oxidized) from cellulose using HPAEC-PAD. Significant amounts of C1- and C4-oxidized oligosaccharides were detected in the early stages of incubation (1, 2, and 3 h), which indicate that several cleavages had already occurred. The concentration of oxidized and non-oxidized oligosaccharides increased gradually up to 48 h with cellobionic acid as the main end product (Fig. 4). At the end of the reaction, around 10 % of cellulose was converted into soluble products (although it was not possible to quantify the concentration of C4-oxidized oligosaccharides). Noteworthy, the concentration of C1-oxidized cellopentaose (DP5ox) declined after 9 h of incubation (Fig. 4), which could indicate a potential action of *Pa*LPMO9H on soluble oligosaccharides in a similar way as was observed for NcLPMO9C [30].

#### P*a*LPMO9H displays broad specificity against cello-oligosaccharides and β-(1,4)-linked polysaccharides

**Fig. 5:**
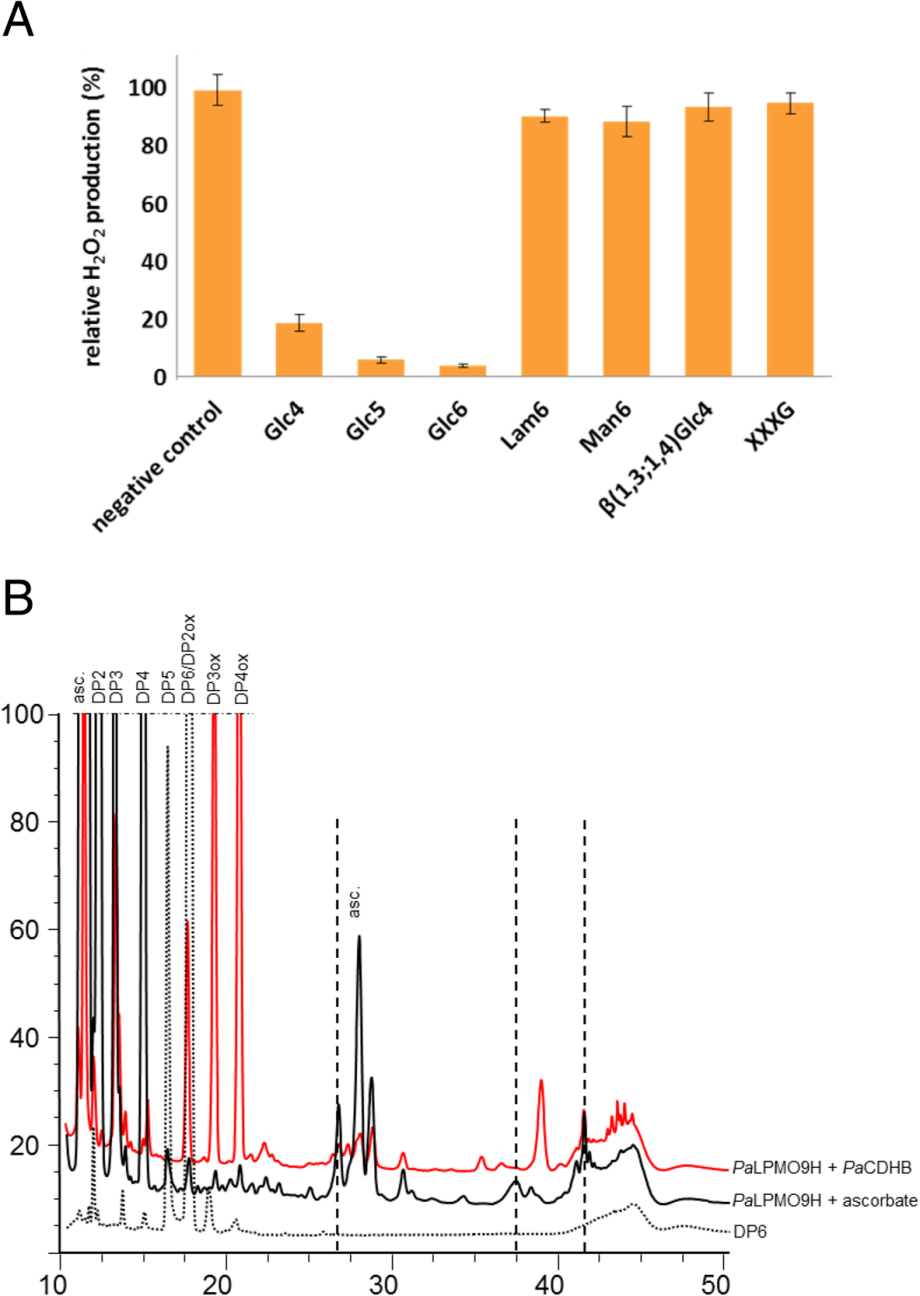
*Pa*LPMO9H activity on oligosaccharide substrates. **a** Generation of H_2_O_2_ by *Pa*LPMO9H in the presence and/or absence of various oligosaccharides substrates. Glc4, cellotetraose; Glc5, cellopentaose; Glc6, cellohexaose; Lam6, laminarinhexaose; Man6, mannohexaose; β(1,3;1,4)Glc4, β(1,3;1,4)-glucotetraose (G4G3G4G); XXXG, xyloglucan-derived heptasaccharide. **b** HPAEC chromatogram of products released from cellohexaose by action of *Pa*LPMO9H in the presence of ascorbic acid (in *black*) or *Pa*CDHB (in *red*) with the same labeling of peaks as Fig. 2

Among the set of *Pa*LPMO9s studied, *Pa*LPMO9H was the only one displaying inhibition of H_2_O_2_ production in the presence of cellohexaose (DP6), cellopentaose (DP5), and cellotetraose (DP4) with the residual H_2_O_2_ production of 4.8, 6.5, and 19.4 %, respectively (Fig. 5a). However, no decrease in H_2_O_2_ production was observed in the presence of non-cellulosic oligosaccharides [mannohexaose, laminarihexaose, xyloglucan-derived heptasaccharide (XXXG), and mixed linkage β-(1-3,1-4)-tetraose] (Fig. 5a) suggesting that *Pa*LPMO9H is not active on these oligosaccharide substrates. To verify these findings, we used the HPAEC method to detect native and oxidized species using cellopentaose and cellohexaose as substrates. In the presence of *Pa*CDHB or ascorbic acid, *Pa*LPMO9H was able to oxidatively cleave cellohexaose since the peak decreased significantly with the concomitant apparition of oxidized and non-oxidized species (Fig. 5b). The ascorbate condition yielded the release of C1-oxidized oligosaccharides (DP3ox and DP4ox) as well as presumably C4-oxidized species observed at 27 and 38 min (Fig. 5b). The use of *Pa*CDHB as a donor of electron led to an increase of C1-oxidized oligosaccharides and to the apparition of a major peak eluting at 39 min which may correspond to a double-oxidized DP2 (C1–C4) and to longer double-oxidized products (C1–C4) eluting around 42–44 min (Fig. 5b) as observed for cellulose (Fig. 2). A similar pattern of products was obtained when cellopentaose was used as substrate (Additional file 1: Figure S4).

**Fig. 6:**
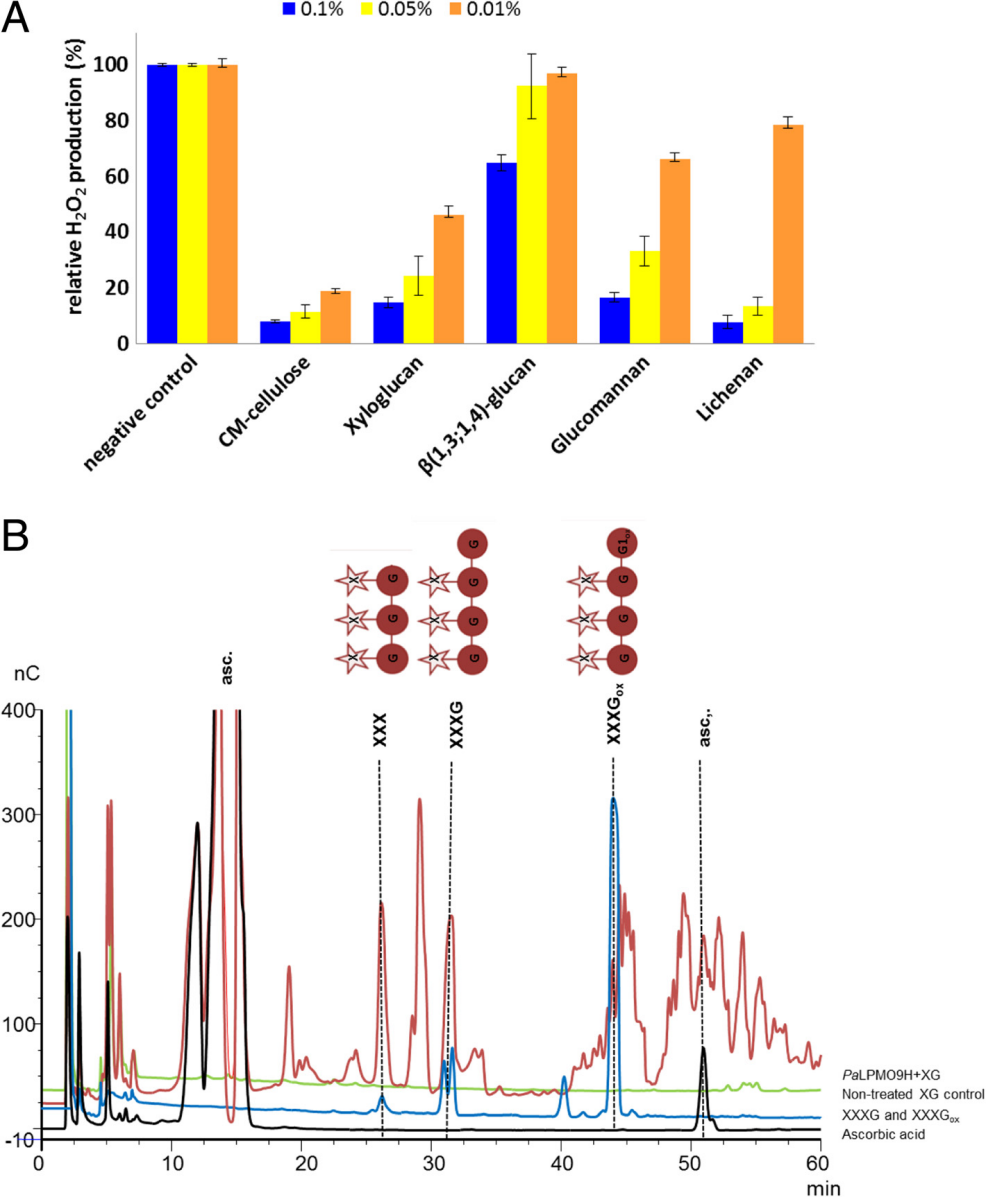
*Pa*LPMO9H activity on polysaccharide substrates. **a** Relative production of H_2_O_2_ by *Pa*LPMO9H in the presence and/or absence of various polysaccharides substrates. **b** HPAEC chromatogram of products released from xyloglucan (XG) by action of *Pa*LPMO9H in the presence of ascorbic acid. The C1-oxidized xyloglucan-derived heptasaccharide standard (XXXGox in *blue*) was prepared from XXXG (based on the nomenclature defined by [49]) using the *Pa*CDHB as described in Material and Methods

To further investigate the substrate specificities of *Pa*LPMO9s, we used the method based on H_2_O_2_ detection to rapidly screen the ability of *Pa*LPMO9s to interact with a range of potential hemicellulosic substrates. We investigated the differences in H_2_O_2_ production in the presence of CMC, curdlan, barley β-glucan, glucomannan, lichenan, pectin, xylan, and xyloglucan (XG) at three different concentrations ranging from 0.01 to 0.1 % (w/v). The repression in H_2_O_2_ production tested at different substrate concentrations was significant only for *Pa*LPMO9H in the presence of CMC, barley β-glucan, glucomannan, lichenan, and XG (Fig. 6a) while curdlan, pectin, and xylan had no effect on H_2_O_2_ production for any of the *Pa*LPMO9s tested (not shown). The oxidative cleavage of XG was further investigated using HPAEC. It revealed that a mixture of non-oxidized oligosaccharides, some of which match XXX and XXXG species, eluting at 26 and 31 min, respectively. Several other species eluting at later retention times between 40 and 60 min may correspond to C1- and C4-oxidized species [9] (Fig. 6b).

## Discussion

Although the discovery of LPMOs attracted considerable attention due to their beneficial use in biomass conversion to biofuels, to date, only 14 fungal AA9 LPMOs have been biochemically characterized [4–10, 14, 31, 36]. In the present study, we selected six novel LPMOs and successfully characterized in depth three of them (*Pa*LPMO9A, *Pa*LPMO9E, and *Pa*LPMO9H) due to their higher capacity of cellulose cleavage. Among them, the absence of methylation of the N-terminal histidine in *Pa*LPMO9s heterologously expressed in *P. pastoris* was not detrimental for their functionality.

Approximately 20 % of the fungal AA9 LPMOs possess a C-terminal CBM1 specific for cellulose [3], and in the genome of *P. anserina*, out of the 33 LPMOs, five harbor a CBM1 [26]. In this study, we revealed that modular *Pa*LPMO9s with anchored CBM1 release more oxidized oligosaccharides from cellulose than CBM1-less LPMOs. It is generally acknowledged that CBM1 potentiate the action of CAZymes including cellulases [29], hemicellulases [37], and oxidoreductases [38]. Indeed, carbohydrate-binding modules (CBMs) target their appended catalytic domain to the insoluble substrate and increase the enzyme local concentration [39–41]. Recently, bacterial AA10 LPMOs integrated into designer cellulosomes were shown to promote overall cellulose degradation [42]. Therefore, the presence of CBMs in LPMOs is an important feature that should be considered for the design of enzyme cocktails for biomass deconstruction purposes.

Three regioselective groups of AA9 LPMOs (types 1, 2 and 3) have been established based on sequence alignments [8, 12]. In this study, we showed that *Pa*LPMO9A and *Pa*LPMO9E belong to type 3 (oxidation at both the C1 and C4 positions) and type 1 (oxidation at the C1 position only), respectively, in good agreement with the prediction made based on phylogenetic analyses [12]. However, we also evidenced using ionic chromatography and mass spectrometry that *Pa*LPMO9H behaved as a type 3 LPMO (oxidation at the C1 and C4 positions) although from the sequence, it was predicted as a type 2 LPMO (oxidation at the C4 position only) [12]. This classification based on alignments contains other exceptions since a subgroup of type 3 LPMOs (PMO3*) was recently created because the *Myceliophtora thermophila* AA9 LPMO (MYCTH_92668) was shown to oxidize at C1 position only [12]. The type 1, type 2, and type 3 classification based on the phylogenetic analysis using the entire amino acid sequence of AA9 catalytic modules might not be adapted to confidently predict AA9 LPMOs regioselectivity since only amino acids interacting directly with the substrate should be considered, but unfortunately so far, no 3D structure of AA9 LPMOs in complex with their substrate has been solved. Interestingly, AA9 protein sequences were divided into 16 subfamilies by finding short and conserved peptide motifs, but due to the low sequence similarity within the AA9 family, these subfamilies do not help to predict the substrate regioselectivity of AA9 LPMOs [43]. Meanwhile, further biochemical characterization is required to strengthen the prediction of AA9 LPMOs regioselectivity.

Time-course analysis of cellulose cleavage revealed that the dual action of *Pa*LPMO9H and *Pa*CDHB were able to release soluble products at the early stage of the reaction. This result indicates that several oxidative cleavages occurred quite rapidly with the action of *Pa*LPMO9H that released C4- and C1-oxidized species as well as non-oxidized species that are further oxidized at C1 position by *Pa*CDHB.

We further clearly evidenced that *Pa*LPMO9H was able to cleave cello-oligosaccharides using both H_2_O_2_ repression assays and ionic chromatography. *Pa*LPMO9H displayed affinity for non-cellulosic substrates including XG, β-glucans, and glucomannan. This broad substrate specificity towards cellodextrins and hemicellulose is not due to the presence of a CBM1 because *Pa*LPMO9A and *Pa*LPMO9E that also harbor a CBM1 did not display any activity on β-(1,4)-linked polysaccharides other than cellulose. Therefore, the minimal substrate requirement for *Pa*LPMO9H is the presence of β-(1,4)-linked glucose in the backbone. In this regard, *Pa*LPMO9H is similar to *Nc*LPMO9C from *Neurospora crassa* which has been recently shown to cleave oxidatively cello-oligosaccharides [30] as well as hemicellulose polysaccharides such as XG [9]. The main difference relies on the fact that *Nc*LPMO9C cleaves only at the C4 position whereas *Pa*LPMO9H cleaves at both the C1 and C4 positions (although it seems to be predominantly at position C4). Therefore, the pattern of products obtained is different to *Nc*LPMO9C with a broader diversity of product species released in the case of *Pa*LPMO9H.

In the structural model of *Nc*LPMO9C built using the crystal structure of *Nc*LPMO9D (PDB 4EIR [44]), three asparagine residues exposed at the surface of *Nc*LPMO9C were suggested to be involved in the interaction with oligosaccharides, constituting the +2 subsite [30]. In *Pa*LPMO9H, the three asparagine residues (Asn25, Asn26, Asn27) are substituted by Ser25, Asn26, and Phe27 (Additional file 1: Figure S1). Thus, the presence of the three asparagine residues may not be strictly required for the binding of oligosaccharides. *Pa*LPMO9H is the only AA9 LPMO to bear a phenylalanine residue at this position (Phe27) that would probably interact with the sugar through stacking interactions. Therefore, it is tempting to speculate that a strong +2 subsite is a prerequisite of LPMO binding to oligosaccharides. The presence of a phenylalanine residue in *Pa*LPMO9H may also explain its higher affinity for DP4 (Fig. 5a) compared to *Nc*LPMO9C [30] and also the presence of oxidized DP2 products using cellopentaose or cellohexaose as substrate.

Concerning *Pa*LPMO9H specificity for XG, it is interesting to note the amino acids spanning the loop from Gly64 to Ser83 are also found in *Nc*LPMO9C that is to date the only AA9 LPMO displaying XG specificity [9]. This loop exposed at the surface in the structural model of *Nc*LPMO9C [9] is a peculiarity of *Pa*LPMO9H and *Nc*LPMO9C as compared to other characterized fungal AA9 LPMOs. Although XG activity has not been assessed for all the LPMOs studied to date, it is tempting to speculate that these loop-bearing charged residues (Glu66, Asp75, and Asp77) might be a prerequisite for XG specificity.

## Conclusions

*P. anserina* represents an interesting model to study the oxidative deconstruction of lignocellulose since this coprophilous fungus displays an impressive array of genes encoding putative AA9 LPMOs. In this study, we start to unveil the reason for the existence of multiple AA9 LPMOs secreted naturally by this fungus. Indeed, the three enzymes characterized in depth have a different role by targeting different components of the plant cell wall (cellulose, soluble oligosaccharides, and hemicellulose) and/or generating different oxidized and non-oxidized products. In the present study, we clearly demonstrated that the broad substrate specificity of AA9 LPMOs towards oligosaccharides and non-cellulosic polysaccharides is not unique to *N. crassa*. It opens new prospects concerning the biological role of LPMOs in the degradation and modification of non-recalcitrant plant cell wall polysaccharides.

## Material and methods

### Cloning and production of *P. anserina* LPMO9s and CDH

The cloning of *lpmo9A* gene from *P. anserina* strain S mat^+^, encoding *Pa*LPMO9A (protein ID CAP73254), was described by Bey et al. [14] Genes encoding *Pa*LPMOC (protein ID CAP68173), *Pa*LPMO9D (protein ID CAP66744), *Pa*LPMO9E (protein ID CAP67740), *Pa*LPMO9F (protein ID CAP71839), *Pa*LPMO9G (protein ID CAP73072), and *Pa*LPMO9H (protein ID CAP61476) were codon optimized for *P. pastoris* (GenScript, Piscataway, USA) and further inserted into the vector pPICZαA (Invitrogen, Cergy-Pontoise, France) using XhoI and XbaI restriction sites in frame with the (His)_6_ tag (located at the C terminus of recombinant proteins). *P. pastoris* strain X33 and the pPICZαA vector are components of the *P. pastoris* Easy Select Expression System (Invitrogen). All media and protocols are described in the *Pichia* expression manual (Invitrogen). Recombinant expression plasmids were sequenced to check the integrity of the corresponding sequences. Transformation of competent *P. pastoris* X33 was performed by electroporation with SacI-linearized pPICZαA recombinant plasmids as described in [45]. Zeocin-resistant *P. pastoris* transformants were then screened for protein production. The best-producing transformant was grown in a 1 l of BMGY containing 1 ml.l^−1^ of *Pichia* trace minerals 4 (PTM_4_) salts (2 g.l^−1^ CuSO_4_.5H_2_O, 3 g.l^−1^ MnSO_4_.H_2_O, 0.2 g.l^−1^ Na_2_MoO_4_.2H_2_O, 0.02 g.l^−1^ H_3_BO_3_, 0.5 g.l^−1^ CaSO_4_.2H_2_O, 0.5 g.l^−1^ CaCl_2_, 12.5 g.l^−1^ ZnSO_4_.7H_2_O, 22 g.l^−1^ FeSO_4_.7H_2_O, biotin 0.2 g.l^−1^, H_2_SO_4_ 1 ml.l^−1^) in shaken flasks at 28 °C in an orbital shaker (200 rpm) for 16 h to an OD_600_ of 2–6. Expression was induced by transferring cells into 200 ml of BMMY containing 1 ml.l^−1^ of PTM_4_ salts at 20 °C in an orbital shaker (200 rpm) for another 3 days. Each day, the medium was supplemented with 3 % (v/v) methanol. Bioreactor production of the best-producing transformant was carried out in a 2-l bioreactor Tryton (Pierre Guerin, Mauze, France) according to the *P. pastoris* fermentation process guidelines (Invitrogen).

### Enzyme purification

After harvesting cells by centrifugation, the supernatant was dialyzed against buffer A (Tris-HCl 50 mM pH 7.8, NaCl 150 mM, imidazole 10 mM) and loaded onto a His-Trap Resin (GE Healthcare, Buc, France) column (1.60 × 2.50 or 1.60 × 10 cm) equilibrated with buffer A that was connected to an Äkta purifier 100 (GE Healthcare). (His)_6_-tagged recombinant enzymes were eluted with buffer B (Tris-HCl 50 mM pH 7.8, NaCl 150 mM, imidazole 500 mM). Fractions containing recombinant enzymes were pooled, concentrated, and dialyzed against sodium acetate buffer 50 mM, pH 4.8.

### Protein analysis

Proteins were loaded onto 10 % SDS-PAGE gels (Thermo Fisher Scientific) and stained with Imperial Protein Stain (Thermo Fisher Scientific, IL, USA). The molecular mass under denaturating conditions was determined with reference standard proteins (PageRuler Prestained Protein Ladder, Thermo Fisher Scientific). Protein concentration was determined by using the Bradford assay (Bio-Rad, Marnes-la-Coquette, France). Tryptic digest of each purified recombinant protein was analyzed using MS as described in [27].

#### Enzyme assays

The activity of CDH was determined by monitoring the reduction of 0.2 mM 2,6-DCPIP in 100 mM sodium acetate buffer (pH 4.8) containing 10 mM cellobiose as described in [46].

#### Amplex red assay

A fluorimetric assay based on Amplex Red and horseradish peroxidase was used as described previously [30, 31]. The reaction (total volume 100 μl, 30 °C, 30 min) was measured in 100 mM sodium acetate buffer pH 6.0 containing 50 μM Amplex Red (Sigma-Aldrich, Saint-Quentin Fallavier, France), 7.1 U.ml^−1^ horseradish peroxidase, 0.2 to 4 μM *Pa*LPMO9, and 50 μM ascorbate as reductant in water and fluorescence and was detected using an excitation wavelength of 560 nm and an emission wavelength of 595 nm using a Tecan Infinite M200 plate reader (Tecan, Männedorf, Switzerland). The specific activity was counted from H_2_O_2_ calibration curve, and the slope (13,227 counts μmol^−1^) was used to convert the fluorimeters’ readout (counts min^−1^) into enzyme activity. For inhibition studies, the range of polysaccharides (cello-oligosaccharides DP4-DP6, PASC, CMC, β(1,3;1,4)-glucan from barley, β(1,3)-glucan, lichenan, starch, glucomannan, laminarin, pectin, xylan, and XG) and their cello-oligosaccharides derivatives were added to a final concentration of 0.1 % (w/v) and 3 mM, respectively. All measurements were performed in triplicates.

### Cellulose, xyloglucan, and cello-oligosaccharide cleavage assays

All the cleavage assays (300 μl liquid volume) contained 4.4 μM of *Pa*LPMO9s, 1.2 U.ml^−1^ of *Pa*CDHB or 1 mM of ascorbate, and 0.1 % (w/v) PASC prepared from Avicel as described by [47] in 50 mM sodium acetate buffer pH 4.8 or 50 μM of cello-oligosaccharides (Megazyme, Wicklow, Ireland) in 10 mM sodium acetate buffer pH 4.8. The enzyme reactions were performed in 2-ml tubes and incubated in a thermomixer (Eppendorf, Montesson, France) at 50 °C and 850 rpm. After 16 h of incubation, all the samples were boiled at 100 °C for 10 min to stop the enzymatic reaction and then centrifuged at 16,000 rpm for 15 min at 4 °C to separate the soluble fraction from the remaining insoluble fraction before carbohydrate determination. For kinetic experiments, reactions were run as described above and stopped after 1, 2, 3, 5, 7, 9, 24, 30, and 48 h of incubation. Assays were performed as triplicate independent experiments. For XG, the reaction mixture (300 μl liquid volume) contained 4.4 μM of *Pa*LPMO9H, 1 mM of ascorbate, and 0.2 % (w/v) tamarind XG (Megazyme) in 50 mM sodium acetate buffer pH 4.8. The enzyme reactions were performed in 2-ml tubes and proceed as described above. Assays were performed as triplicate independent experiments.

### Analysis of oxidized and non-oxidized oligosaccharides

Mono-, oligosaccharides and their corresponding aldonic acid forms generated after PASC, oligosaccharides, and XG cleavage were analyzed by ionic chromatography (HPAEC) as described by [9] and [32] using non-oxidized oligosaccharides (Megazyme) as standards. Corresponding C1-oxidized standards (from DP2 to DP6) were produced from non-oxidized cello-oligosaccharides by *Pa*CDHB treatment. All assays were carried out in triplicate.

### Mass spectrometry

Products resulting from enzyme reaction in water were also analyzed by MS and two types of mass measurements were performed on the samples: firstly, a mass profile was done by matrix-assisted laser desorption/ionization (MALDI)-time-of-flight (TOF) MS; ions of interest were further fragmented by collision-induced dissociation on an ESI MS/MS instrument. For MALDI-TOF MS measurements, an ionic preparation of 2,5-dihydroxybenzoic acid (DHB) and N,N-dimethylaniline (DMA) was used as the MALDI matrix, as described in [48]. Briefly, the matrix consists of an equimolar mixture of DHB and DMA (DHB 100 mg.ml^−1^, in H_2_O/acetonitrile/DMA (1:1:0.02)) and was mixed with the samples in a 1:1 ratio (v/v), and the mixture (1 μL) was deposited on a polished steel MALDI target plate. MALDI measurements were then performed on an Autoflex Speed MALDI‐TOF/TOF spectrometer (Bruker Daltonics, Bremen, Germany) equipped with a Smartbeam laser (355 nm, 200 Hz) and controlled using the Flex Control 3.0 software package. The mass spectrometer was operated with positive polarity in a reflectron mode, and spectra were acquired in the range of 500–2000 m/z. For ESI MS/MS measurements, experiments were performed on a Synapt G2Si high-definition mass spectrometer (Waters Corp., Manchester, UK). Samples were diluted 100-fold in MeOH/H_2_0 (1:1, v/v) and infused at 5 μL.min^−1^ in the instrument. The instrument was operated in a positive ionization mode in the so-called sensitivity mode, with an ESI capillary voltage of 3 kV and a sampling cone voltage of 100 V. Fragmentation was done by collision-induced dissociation in the transfer cell of the instrument, using appropriate collision energies depending on the precursor. Data acquisition was carried out using MassLynx software (V4.1) over a mass range of 100–1500 m/z.

## Additional file

Additional file 1:
**Functional characterization of a set of fungal AA9 lytic polysaccharide monooxygenases secreted by** ***Podospora anserina.*** Table S1. Amino-acid identities of the *Pa*LPMO9s studied. Table S2. Identification of the N-terminal peptides bearing the first histidine residue using LC-MS/MS. Figure S1. Multiple sequence alignment of 17 LPMO9s including 12 sequences of characterized LPMO9s and the *Pa*LPMO9s characterized in this study. Figure S2. Electrophoretic analyses of purified recombinant *Pa*LPMO9D, *Pa*LPMO9E, *Pa*LPMO9F, *Pa*LPMO9G, *Pa*LPMO9H and *Pa*CDHB enzymes. Figure S3. Mass spectrometry analysis of degradation products generated from PASC by *Pa*LPMO9E in the presence of ascorbic acid. Figure S4. HPAEC chromatogram of products released from cellopentaose by action of *Pa*LPMO9H in the presence of ascorbic acid or *Pa*CDHB.

## Abbreviations

AA: auxiliary activity enzyme
CBM: carbohydrate-binding module
CDH: cellobiose dehydrogenase
CMC: carboxymethyl cellulose
DHB: 2,5-dihydroxybenzoic acid
DMA: N,N-dimethylaniline
DP: degree of polymerization
LPMO: lytic polysaccharide monooxygenase
MS: mass spectrometry
*Pa*LPMO9: *Podospora anserina* AA9 LPMO
PASC: phosphoric acid swollen cellulose
XG: xyloglucan
XXXG: xyloglucan-derived heptasaccharide
