# Supplementary material for: Substrate specificity and regioselectivity of fungal AA9 lytic polysaccharide monooxygenases secreted by Podospora anserina
Source: Biotechnol Biofuels. 2015 Jun 20;8:90. doi: 10.1186/s13068-015-0274-3 (PMC4487207; doi:10.1186/s13068-015-0274-3)

**Supplementary data**

Functional characterization of a set of fungal AA9 lytic polysaccharide monooxygenases secreted by *Podospora anserina*

Chloé Bennati-Granier^1,2,†^, Sona Garajova^1,2,3,†^, Charlotte Champion^1,2^, Sacha Grisel^1,2^, Mireille Haon^1,2^, Simeng Zhou^1,2^, Mathieu Fanuel^4^, David Ropartz^4^, Hélène Rogniaux^4^, Isabelle Gimbert^1,2^, Eric Record^1,2^, Jean-Guy Berrin^1,2,*^

Table S1. Amino-acid identities of the *Pa*LPMO9s studied. Alignments were performed using <http://www.ebi.ac.uk/Tools/msa/clustalw2/> using the catalytic domains of *Pa*LPMO9s. Results are expressed in %.

|  | | *Pa*LPMO9A | *Pa*LPMO9D | | *Pa*LPMO9E | | *Pa*LPMO9F | | | *Pa*LPMO9G | | *Pa*LPMO9H | |
| --- | --- | --- | --- | --- | --- | --- | --- | --- | --- | --- | --- | --- | --- |
| *Pa*LPMO9A | | 100 |  | |  | |  | | |  | |  | |
| *Pa*LPMO9D | | 52.8 | 100 | |  | |  | | |  | |  | |
| *Pa*LPMO9E | | 24.1 | 24.6 | | 100 | |  | | |  | |  | |
| *Pa*LPMO9F | | 33.5 | 34.8 | | 34.0 | | 100 | | |  | |  | |
| *Pa*LPMO9G | | 23.9 | 25.7 | | 36.4 | | 32.0 | | | 100 | |  | |
| *Pa*LPMO9H | | 22.9 | 29.8 | | 34.0 | | 27.7 | | | 45.5 | | 100 | |
|  | | |  | |  | |  |  | |  | |  | |

Table S2. *In silico* identification of the N-terminal peptides bearing the first histidine residue using LC-MS/MS. Identifications were validated manually as described in material and methods.

|  | sequence | e-value | MH+ obs | MH+ theo |
| --- | --- | --- | --- | --- |
| *Pa*LPMO9E | HYIFQQLSTGSTK | 7.00E-04 | 1509.7706 | 1509.7700 |
|  | HYIFQQLSTGSTK | 6.10E-04 | 1509.7697 | 1509.7700 |
|  | HYIFQQLSTGSTK | 3.70E-07 | 1509.7705 | 1509.7700 |
|  | HYIFQQLSTGSTK | 2.60E-07 | 1509.7704 | 1509.7700 |
| *Pa*LPMO9H | HSIFQKVSVNGVDQGQLK | 8.70E-05 | 1984.0625 | 1984.0615 |

Figure S1. Multiple sequence alignment of 17 LPMO9s including 12 sequences of characterized LPMO9s and the *Pa*LPMO9s characterized in this study. Only the AA9 catalytic module of each sequence was considered and aligned using <http://www.ebi.ac.uk/Tools/msa/clustalw2/>. Asterisks indicate amino-acids coordinating the copper ion. *Pa*LPMO9H amino-acids potentially involved in oligosaccharide binding (Asn26 and Phe27) and xyloglucan binding (from Gly64 to Ser83) are highlighted in bold characters.

MYCTH112089-C1 HYTFPRAGTGGS-LSGEWEVVRMTENH------------YSHGPVTDVTSPEMTCYQS 45

NcLPMO9F-C1 HYTFPKVWANSG-TTADWQYVRRADNW------------QNNGFVDNVNSQQIRCFQS 45

PcLPMO9D-C1 HYTFPDFIEPSGTVTGDWVYVRETQNH------------YSNGPVTDVTSPEFRCYEL 46

NCU00836-C1 HYIFQQVGAGTS-VNPVWKYIRKHTNY--------------NSPVTDLTSKDLVCNVG 43

PaLPMO9E HYIFQQLSTGST-KHGVFQYIRQNTNY--------------NSPVTDLSSNDLRCNEG 43

NcLPMO9D-C4 HTIFSSLEVNGVNQGLGEGVRVP----------------TYNGPIEDVTSASIACNGS 42

PaLPMO9G HTIFSSLEVNGVNHGVGGGVRVP----------------SYNGPIENVDSASIACNGA 42

NCU02240-C4 HTIFVQLEADGTTYPVSYGIRTP----------------SYDGPITDVTSNDLACNGG 42

NcLPMO9C-C4 HTIFQKVSVNGADQGQLKGIRAP----------------ANNNPVTDVMSSDIICN-- 40

PaLPMO9H HSIFQKVSVNGVDQGQLKGVRAP----------------YS**NF**PIENVNHPDFACNT- 41

NcLMPO9E-C1 HATFQALWVDGADYG-SQCARVP----------------PSNSPVTDVTSNAMRCN-- 39

PaLPMO9B-C4 HSTFQQLWVDGTDFG-SQCARLP----------------QSNSPITNYNSNDMRCN-- 39

NcLPMO9M-C1_C4 HGFVDNATIGGQFYQFYQPYQDPYMGS-PPDRISRKIP--GNGPVEDVTSLAIQCN-- 53

PaLPMO9F HGYVDNATIGGQYYQFYQPYMDPYMGNNKPQRVSRSIP--GNGPVENVDSIDVQCN-- 54

NCU07760-C1_C4 HGHVSKVIVNGVEYQNYDPTSFPYNSN-PPTVIGWTIDQKDNGFVSPDAFDSGDIICH 57

PaLPMO9A-C1_C4 HGHVSHIIVNGVYYQNYDPTTHFYQPN-PPTVIGWSALQQDNGFVEPNNFGTTDIICH 57

PaLPMO9D HGHVKNVVVNGASFQGYDINSFPYTQN-PPKVAAWTASNTDNGFVGPESFASSDIICH 57

*****

MYCTH112089-C1 GVQ---GAPQTVQVKAGSQFTFSVDP------------------------SIGHPGPLQF 78

NcLPMO9F-C1 THS---PAQSTLSVAAGTTITYGAAP------------------------SVYHPGPMQF 78

PcLPMO9D-C1 DLQNTAGQTQTATVSAGDTVGFKANS------------------------AIYHPGYLDV 82

NCU00836-C1 -AS--AEGVETLSVAAGSQVTFKTDT------------------------AVYHQGPTSV 76

PaLPMO9E GAS--GANTQTVTVRAGDSFTFHLDT------------------------PVYHQGPVSV 77

NcLPMO9D-C4 -PNTVASTSKVITVQAGTNVTAIWRYMLSTTG-----------DSPADVMDSSHKGPTIA 90

PaLPMO9G -PNPTTPTSKVITVQAGQNVTAIWRYMLSTTG-----------SAPNDIMDISHKGPTMA 90

NCU02240-C4 -PNPTTPSDKIITVNAGSTVKAIWRHTLTSG--------------ADDVMDASHKGPTLA 87

NcLPMO9C-C4 --AVTMKDSNVLTVPAGAKVGHFWGHEIGGAAG---------PNDADNPIAASHKGPIMV 89

PaLPMO9H --NIQLRDNTVIKVPAGARVGAWW**GHEIGGAAG**---------**PNDPDHPIAAS**HKGPIQV 90

NcLMPO9E-C1 --TGTSPVAKKCPVKAGSTVTVEMHQSHPPVPTLTYKQQANDRSCSSEAIGGAHYGPVLV 97

PaLPMO9B-C4 --IIGTRPQVKCPVRAGGTVTVEMHAQN------------GDRSCSQEAIGGAHHGPVSV 85

NcLPMO9M-C1_C4 --ADSAPAKLHASAAAGSTVTLRWT-----------------------IWPDSHVGPVIT 88

PaLPMO9F --AGSVPAPLHAPAAAGSTVTLHWT-----------------------LWPDSHMGPVIT 89

NCU07760-C1_C4 --KSAKPAGGHATVKAGDKISLQWD-----------------------QWPESHKGPVID 92

PaLPMO9A-C1_C4 --KSAAPGGGSATVNAGDKISIVWTP----------------------EWPESHIGPVID 93

PaLPMO9D --KNSANAQGRIVVAAGDSVFVQWD-----------------------TWPESHHGPVID 92

*****

MYCTH112089-C1 YMAKVPSGQTAATFDGTG-AVWFKIYQDGPNG-----------LGTDSITWPSAGKTEVS 126

NcLPMO9F-C1 YLARVPDGQDINSWTGEG-AVWFKIYHEQPT------------FGS-QLTWSSNGKSSFP 124

PcLPMO9D-C1 MMSPASPAANSPE-AGTG-QTWFKIYEEKPQ------------FENGQLVFDTT-QQEVT 127

NCU00836-C1 YLSKADG--SLSDYDGSG-G-WFKIKDWGAT------------FPGGEWTLSDT----YT 116

PaLPMO9E YLSKAPG--SASSYDGSG-T-WFKIKDWGPT------------FPGGQWTLAGS----YT 117

NcLPMO9D-C4 YLKKVDN---AATASGVG-NGWFKIQQDGMDSS-----------GVWGTERVINGKGRHS 135

PaLPMO9G YLKKVND---ATTDSGVG-GGWFKIQEDGYNN------------GVWGTEKVINGQGRHS 134

NCU02240-C4 YLKKVDD---ALTDTGIG-GGWFKIQEDGYNN------------GQWGTSTVITNGGFQY 131

NcLPMO9C-C4 YLAKVDN---AATTGTSG-LKWFKVAEAGLSN------------GKWAVDDLIANNGWSY 133

PaLPMO9H YLAKVNN---AANAGTSG-LQWFKVAEQGLNN------------GVWAVDNMISNGGWHY 134

NcLMPO9E-C1 YMSKVSD---AASADGS--SGWFKIFEDTWAKKPSS---SSGDDDFWGVKDLNSCCGKMQ 149

PaLPMO9B-C4 YLTKVSD---ALTADGS--TGWFKIFDDGWRKNPSG---RVGDDDFWGTKDLNACCGKMN 137

NcLPMO9M-C1_C4 YMARCPDTGCQDWTPSASDKVWFKIKEGGREGT----------SNVWAATPLMTAPANYE 138

PaLPMO9F YMARCPDSGCQNWSPGTS-AVWFKIKQGGREGT----------SNNWAATPLMKSPATYQ 138

NCU07760-C1_C4 YLAACDGD-CESVDKTAL--KFFKIDGAGYDA-----------TNGWASDTLIKDGNSWV 138

PaLPMO9A-C1_C4 YLANCNGP-CETVDKTSL--RWFKIGGAGYNPN----------TRTWAADDLRANGNSWL 140

PaLPMO9D YLASCGNTGCDKIEKTAL--EFFKIAEAGLVNGA-------QAPGRWASDVLIDNNNSWM 143

MYCTH112089-C1 VTIPSCIEDGEYLLRVEHIALHSASSVGGAQFYIACAQLSVTGGSGTLN-TGSLVSLPGA 185

NcLPMO9F-C1 VKIPSCIKSGSYLLRAEHIGLHVAQSSGAAQFYISCAQLSITGGGSTEPGANYKVSFPGA 184

PcLPMO9D-C1 FTIPKSLPSGQYLLRIEQIALHVASSYGGAQFYIGCAQLNVENGGNGTP--GPLVSIPGV 185

NCU00836-C1 FTIPSCIPSGDYLLRIQQIGIHNPWPAGVPQFYLSCAHISVTGGGSASP---ATVSIPGA 173

PaLPMO9E AQLPSCITDGEYLLRIQSLGIHNPYPAGTPQFYISCAQIKVTGGGSVNP---SGVAIPGA 174

NcLPMO9D-C4 IKIPECIAPGQYLLRAEMIALHAASNYPGAQFYMECAQLNVVGGTG--AKTPSTVSFPGA 193

PaLPMO9G IKIPSCIAPGQYLLRAEMLALHGAGNYPGAQFYMECAQLNIVGGTG--SKTPSTVAFPGA 192

NCU02240-C4 IDIPACIPSGQYLLRAEMIALHAASSTAGAQLYMECAQINIVGGTGGTALPSTTYSIPGI 191

NcLPMO9C-C4 FDMPTCIAPGQYLMRAELIALHNAGSQAGAQFYIGCAQINVTGGGS--ASPSNTVSFPGA 191

PaLPMO9H FDMPSCVAPGHYLMRVELLALHSASVRGAAQFYMECAQIEITGSGT--NTGSNFVSFPGA 192

NcLMPO9E-C1 VKIPSDIPAGDYLLRAEVIALHTAASAGGAQLYMTCYQISVTGGGS---ATPATVSFPGA 206

PaLPMO9B-C4 VKIPSDIPSGDYLLRAEAIALHAAGGAGGAQPYMTCYQITVSGGGS---ASPPTVSIPGH 194

NcLPMO9M-C1_C4 YAIPSCLKPGYYLVRHEIIALHSAYSYPGAQFYPGCHQLQVTGSGTKTPSSG-LVSFPGA 197

PaLPMO9F YTIPSCIRPGYYLVRHEIIALHAAWAYPGAQFYPGCHQLQVTGGGSTNPTN--LVSFPGA 196

NCU07760-C1_C4 VEIPESIKPGNYVLRHEIIALHSAGQANGAQNYPQCFNLKVEGSGSTVPAG---VAGTEL 195

PaLPMO9A-C1_C4 VQIPADLKAGNYVLRHEIIALHGGSSPNGAQAYPQCLNLRIVGNGNNSPAG---VAGTSL 197

PaLPMO9D IKIPANIRPGQYVLRHEIIALHSGGDLNGAQNYPQCFNIEVTGSGTVLPQG---VKGTSL 200

*****

MYCTH112089-C1 YKATDPGILFQLYWPIP--T----EYINPGPAPVSC--- 215

NcLPMO9F-C1 YKASDPGILININYPVP--T----SYKNPGPSVFTC--- 214

PcLPMO9D-C1 YTGYEPGILINIYNLPKNFT----GYPAPGPAVWQG--- 217

NCU00836-C1 FKETDPGYTVNIYSNFN-------NYTVPGPEVFTC--- 202

PaLPMO9E FKATDPGYTANIYSNFN-------SYTVPGPSVFSC--- 203

NcLPMO9D-C4 YSGSDPGVKISIY-WPPVTS-----YTVPGPSVFTC--- 223

PaLPMO9G YSGSHPGVKISIY-WPPVTN-----YQIPGPSVFTC--- 222

NCU02240-C4 YKATDPGLLVNIYSMSPSST-----YTIPGPAKFTC--- 222

NcLPMO9C-C4 YSASDPGILINIYGGSGKTDNGGKPYQIPGPALFTCPAG 230

PaLPMO9H YTADHPGILVSIYDLQGRPTNGGRPYTIPGPAPLTC--- 228

NcLMPO9E-C1 YKSSDPGILVDIHSAMST-------YVAPGPAVYSG--- 235

PaLPMO9B-C4 FKASDPGVQVNIHGAMTN-------YVIPGPAVYAG--- 223

NcLPMO9M-C1_C4 YKSTDPGVTYDAYQAAT--------YTIPGPAVFTC--- 225

PaLPMO9F YKSTDPGVTYDAYKAQA--------YTIPGPAVFTC--- 224

NCU07760-C1_C4 YKATDAGILFDIYKNDIS-------YPVPGPSLIAG--- 224

PaLPMO9A-C1_C4 YRANDAGILFNPYVASPN-------YPVPGPALIAGI-- 227

PaLPMO9D YTPTDAGIRFNIYRSLDS-------YPIPGPALPAG--- 229

Figure S2. Electrophoretic analyses of purified recombinant *Pa*LPMO9D, *Pa*LPMO9E, *Pa*LPMO9F, *Pa*LPMO9G, *Pa*LPMO9H and *Pa*CDHB enzymes.


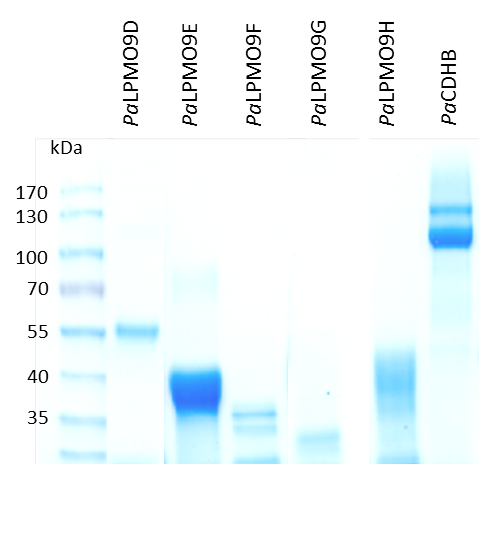


Figure S3. Mass spectrometry analysis of degradation products generated from PASC by *Pa*LPMO9E in the presence of ascorbic acid. Analysis was performed after 16 hours of incubation in water. The panel A shows the full spectrum of the sample with peaks corresponding to native and oxidized cello-oligosaccharides. Panel B shows the ESI MS/MS fragmentation pattern of the DP4 peak (m/z value of 727.22) that corresponds to a C1-oxidized product (sodium adduct of the aldonic acid form).


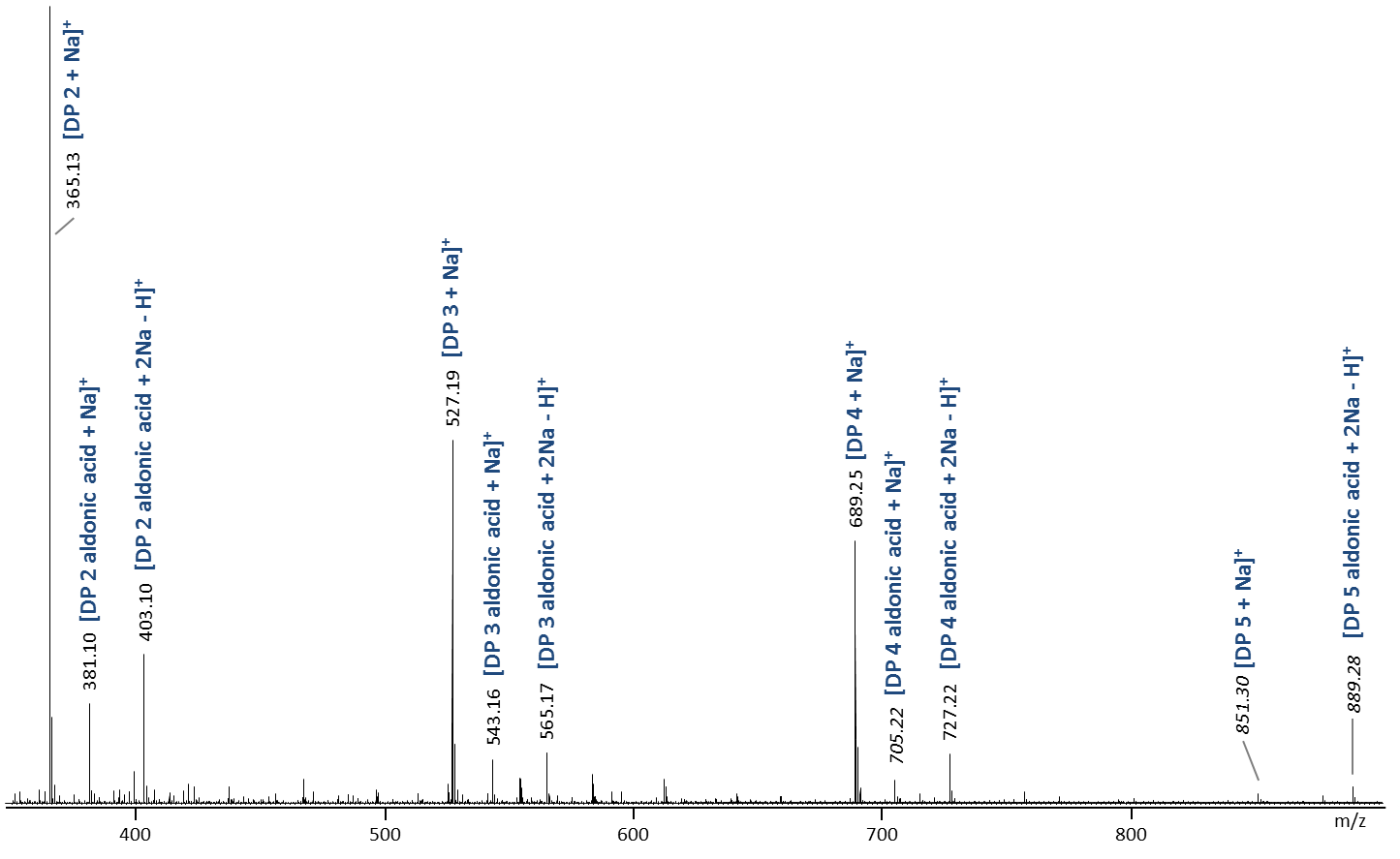


A


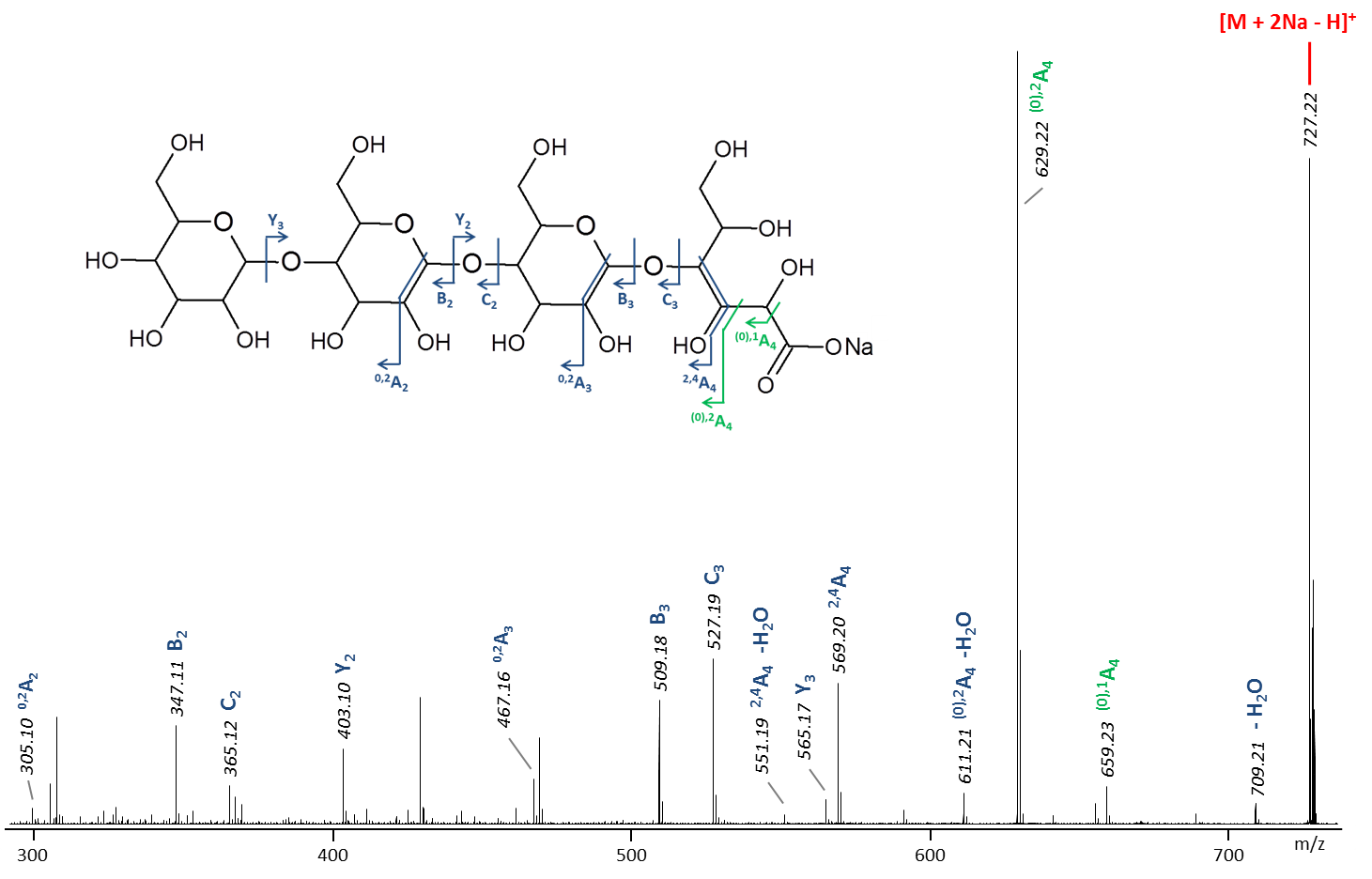


B

Figure S4. HPAEC chromatogram of products released from cellopentaose by action of *Pa*LPMO9H in the presence of ascorbic acid (in black) or *Pa*CDHB (in red). Peaks are labeled according to Fig. 2.


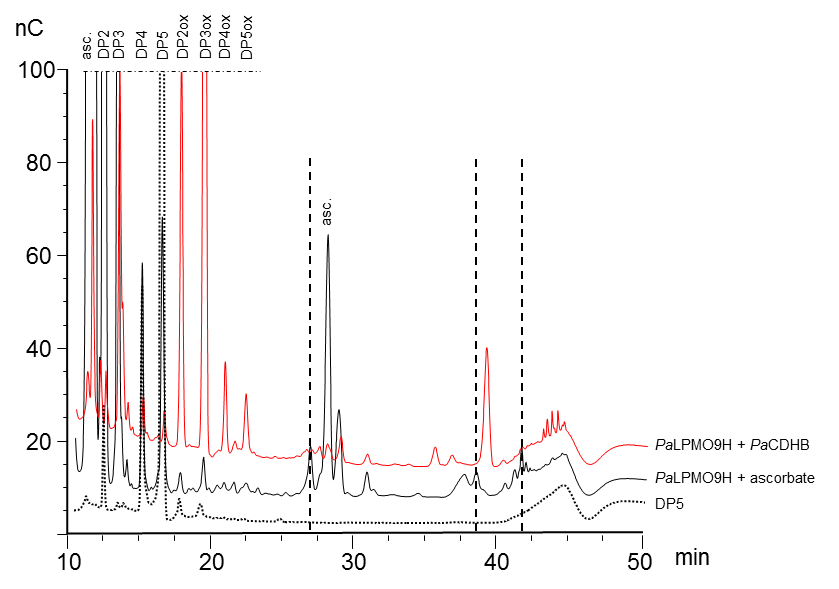

Supplement: Additional file 1: — Functional characterization of a set of fungal AA9 lytic polysaccharide monooxygenases secreted by Podospora anserina. Table S1. Amino-acid identities of the PaLPMO9s studied. Table S2. Identification of the N-terminal peptides bearing the first histidine residue using LC-MS/MS. Figure S1. Multiple sequence alignment of 17 LPMO9s including 12 sequences of characterized LPMO9s and the PaLPMO9s characterized in this study. Figure S2. Electrophoretic analyses of purified recombinant PaLPMO9D, PaLPMO9E, PaLPMO9F, PaLPMO9G, PaLPMO9H and PaCDHB enzymes. Figure S3. Mass spectrometry analysis of degradation products generated from PASC by PaLPMO9E in the presence of ascorbic acid. Figure S4. HPAEC chromatogram of products released from cellopentaose by action of PaLPMO9H in the presence of ascorbic acid or PaCDHB. [file 13068_2015_274_MOESM1_ESM.docx]
